# GLP-1 Receptor Agonists and Colorectal Cancer Risk in Drug-Naive Patients With Type 2 Diabetes, With and Without Overweight/Obesity

**DOI:** 10.1001/jamaoncol.2023.5573

**Published:** 2023-12-07

**Authors:** Lindsey Wang, William Wang, David C. Kaelber, Rong Xu, Nathan A. Berger

**Affiliations:** 1 Center for Science, Health, and Society, Case Western Reserve University School of Medicine, Cleveland, Ohio; 2 Departments of Internal Medicine, Pediatrics, and Population and Quantitative Health Sciences and the Center for Clinical Informatics Research and Education, The MetroHealth System, Cleveland, Ohio; 3 Center for Artificial Intelligence in Drug Discovery, Case Western Reserve University School of Medicine, Cleveland, Ohio; 4 Case Comprehensive Cancer Center, Case Western Reserve University School of Medicine, Cleveland, Ohio

## Abstract

This cohort study compares glucagon-like peptide 1 receptor agonists (GLP-1RAs) with 7 non–GLP-1RA antidiabetics among drug-naive patients with type 2 diabetes.

Glucagon-like peptide 1 receptor agonists (GLP-1RAs) are approved by the US Food and Drug Administration for treating type 2 diabetes (T2D). GLP-1RAs have pleiotropic effects on lowering plasma glucose, inducing weight loss, and modulating immune functions.^1^ Because overweight/obesity is a major risk factor for colorectal cancer (CRC),^2^ we hypothesize that GLP-1RAs are associated with a decreased risk for CRC in patients with T2D compared with non–GLP-1RA antidiabetics. We conducted a nationwide, retrospective cohort study among drug-naive patients with T2D comparing GLP-1RAs with 7 non–GLP-1RA antidiabetics, including metformin and insulin, which are suggested to influence CRC risk.^3^

## Methods

We used the TriNetX platform to access deidentified electronic health records of 101.2 million patients, including 7.4 million with T2D from 59 health care organizations across 50 states.^4^ TriNetX built-in analytic functions allow for patient-level analyses while only reporting population-level data. The MetroHealth System institutional review board determined that using data from TriNetX is not human subject research and therefore exempt from approval. The TriNetX platform has been shown to be useful for retrospective cancer cohort studies.^5,6^

The study population comprised 1 221 218 patients with T2D who had medical encounters for T2D and were subsequently prescribed antidiabetic medications from 2005 to 2019, no prior antidiabetic medication use (drug naive), and no prior CRC diagnosis. GLP-1RAs were compared with insulin, metformin, alpha-glucosidase inhibitors, dipeptidyl-peptidase 4 (DPP-4) inhibitors, sodium-glucose cotransporter-2 (SGLT2) inhibitors, sulfonylureas, and thiazolidinediones. The time of 2005 to 2019 (except for a starting year of 2013 for SGLT2 inhibitors and 2006 for DPP-4 inhibitors) was chosen based on the year drugs were first approved. The study population was divided into exposure and comparison cohorts for each comparison.

Cohorts were propensity score matched (1:1, using nearest neighbor greedy matching) for demographics, adverse socioeconomic determinants of health, preexisting medical conditions, family and personal history of cancers and colonic polyps, lifestyle factors (exercise, diet, smoking, and alcohol drinking), and procedures such as colonoscopy^2^ (Table). The outcome was the first diagnosis of CRC that occurred within 15 years starting from the index event (first prescription of GLP-1RAs vs non–GLP-1RA antidiabetics). With censoring applied, Kaplan-Meier analysis with hazard ratios (HRs) and 95% CIs were used to compare time to event rates at daily time intervals. Separate analyses were performed in patients stratified by the status of obesity/overweight and sex but not by age groups and race and ethnicity due to limited sample sizes. Data were collected and analyzed on September 13, 2023, within the TriNetX Analytics Platform using built-in functions (R, version 4.0.2 [R Project for Statistical Computing]), with statistical significance set at a 2-sided *P* < .05. More details are available in the eMethods in Supplement 1.

**Table.** Demographic Characteristics of the Cohorts Before Propensity Score Matching^a^

Characteristic	%^b^			
	GLP-1RA(+)/insulin(−) (n = 22 575)	Insulin (+)/GLP-1RA(−) (n = 990 239)	GLP-1RA(+)/metformin(−) (n = 18 520)	Metformin (+)/GLP-1RA(−) (n = 845 150)
Age at index event, mean (SD), y	55.6 (12.3)	60.7 (16.3)	58.2 (12.9)	58.8 (14.2)
Sex				
Female	56.0	46.8	57.3	48.6
Male	42.6	52.7	41.4	50.8
Ethnicity				
Hispanic/Latinx	8.7	9.6	7.5	10.7
Not Hispanic/Latinx	65.1	59.5	66.8	61.3
Unknown	26.3	30.9	25.6	28.0
Race				
American Indian or Alaska Native	0.4	0.5	0.02	0.4
Asian	2.3	4.4	1.9	4.2
Black	12.2	17.7	14.7	16.7
Native Hawaiian or Other Pacific Islander	0.5	1.1	0.08	0.5
White	70.8	61.2	69.8	61.5
Unknown	13.9	15.0	12.7	16.7

Abbreviation: GLP-1RA, glucagon-like peptide 1 receptor agonist.

^a^ The status of variables was based on the presence of related clinical codes anytime to 1 day before the index event. Other variables that were not shown but propensity score matched between cohorts include adverse socioeconomic determinants of health (eg, housing and economic circumstance, upbringing, education, physical environment, social environment), family circumstance, family and personal history of cancers, family and personal history of colonic polyps, family history of colorectal cancer, lifestyle factors (eg, exercise, diet, smoking, alcohol drinking), preexisting medical conditions and procedures such as benign neoplasm of the colon and rectum, overweight and obesity, Crohn disease, ulcerative colitis, cystic fibrosis, bariatric surgery, colonoscopy, and radiation therapy.

^b^ A plus sign (+) indicates that a patient was prescribed a GLP-1RA or non–GLP-1RA antidiabetic medication, while a minus sign (−) indicates that they were not.

## Results

During a 15-year follow-up in 1 221 218 drug-naive patients with T2D, GLP-1RAs were associated with decreased risk for CRC compared with insulin (HR, 0.56; 95% CI, 0.44-0.72), metformin (HR, 0.75; 95% CI, 0.58-0.97), SGLT2 inhibitors, sulfonylureas, and thiazolidinediones, and with lower but not statistically significant risk compared with alpha-glucosidase or DPP-4 inhibitors (Figure, A). Consistent findings were observed in women and in men. GLP-1RAs were associated with a lower risk for CRC in patients with obesity/overweight compared with insulin (HR, 0.50; 95% CI, 0.33-0.75), metformin (HR, 0.58; 95% CI, 0.38-0.89), or other antidiabetics (Figure, B).

**Figure.**
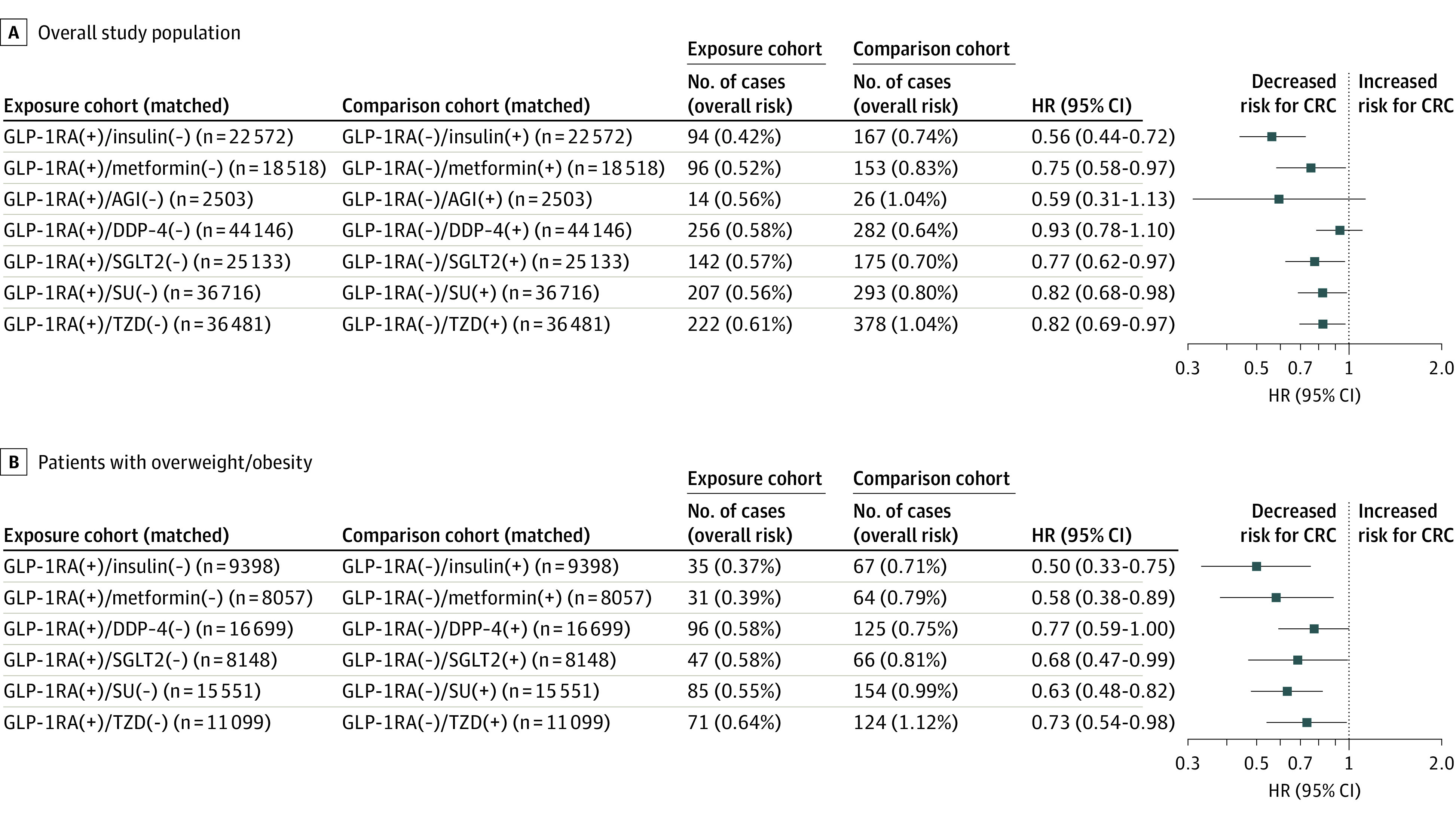
Risks and Hazard Ratios (HRs) of First-Time Diagnosis of Colorectal Cancer (CRC) in Drug-Naive Patients With Type 2 Diabetes Patients had no prior CRC and had no prior antidiabetic medication prescriptions between matched cohorts in the overall study population (A) and in patients with obesity/overweight (B). Kaplan-Meier analysis was used to estimate the probability of the outcome (first diagnosis of CRC) at daily time intervals with censoring applied within a 15-year time window starting from the index event (first prescription of glucagon-like peptide 1 receptor agonists [GLP-1RAs] vs other non–GLP-1RA antidiabetic medications). The cohorts were propensity score matched for demographics, adverse socioeconomic determinants of health, preexisting medical conditions, personal and family history of cancers such as CRC and colonic polyps, benign neoplasms of the colon and rectum, lifestyle factors (exercise, diet, smoking, and alcohol drinking), medical encounters, and procedures such as colonoscopy. Overall risk is defined as the number of incidence cases among the number of patients in each cohort at the beginning of the time window. A plus sign (+) indicates that a patient was prescribed a GLP-1RA or non–GLP-1RA antidiabetic medication, while a minus sign (−) indicates that they were not. AGI indicates alpha-glucosidase inhibitors; DPP-4, dipeptidyl-peptidase 4 inhibitors; SGLT2, sodium-glucose cotransporter-2 inhibitors; SU, sulfonylureas, TZD, thiazolidinediones.

## Discussion

In this cohort study, GLP-1RAs were associated with reduced CRC risk in drug-naive patients with T2D with and without obesity/overweight, with more profound effects in patients with obesity/overweight, suggesting a potential protective effect against CRC partially mediated by weight loss and other mechanisms not related to weight loss. Study limitations include potential unmeasured or uncontrolled confounders, self-selection, reverse causality, and other biases inherent in observational studies, and that results need validation from other data and study populations. Further research is warranted to investigate the effects in patients with prior antidiabetic treatments, underlying mechanisms, potential differential effects within GLP-1RAs, and effects of GLP-1RAs on other obesity-associated cancers.

## Notes

Supplement 1.
eMethods

Supplement 2. Data Sharing Statement
