# Supplementary material for: GLP-1 Receptor Agonists and Colorectal Cancer Risk in Drug-Naive Patients With Type 2 Diabetes, With and Without Overweight/Obesity
Source: JAMA Oncol. 2023 Dec 7;10(2):256–8. doi: 10.1001/jamaoncol.2023.5573 (PMC10704339; doi:10.1001/jamaoncol.2023.5573)
Supplement: Supplement 1. — eMethods [file jamaoncol-e235573-s001.pdf]

Supplemental Online Content

Wang L, Wang W, Kaelber DC, Xu R, Berger NA. GLP-1 receptor agonists and colorectal cancer risk in drug-naive patients with type 2 diabetes, with and without overweight/obesity. *JAMA Oncol*. Published online December 7, 2023. doi:10.1001/jamaoncol.2023.5573

eMethods

This supplemental material has been provided by the authors to give readers additional information about their work.

© 2023 Wang L et al. *JAMA Oncol.*

eMethods

1. Data

The data used in this study were collected and analyzed on September 13, 2023 from the TriNetX "Research US Collaborative Network". We used the TriNetX platform to access aggregated and de-identified electronic health records (EHRs) of 101.2 million patients from 59 healthcare organizations in the US across 50 states, covering diverse geographic regions, age, race/ethnic, income and insurance groups, and clinical settings. TriNetX, LLC is compliant with the Health Insurance Portability and Accountability Act (HIPAA). Any data displayed on the TriNetX Platform in aggregate form, or any patient-level data provided in a data set generated by the TriNetX Platform only contains de-identified data as per the de-identification standard defined in Section §164.514(a) of the HIPAA Privacy Rule. TriNetX built-in analytic functions (e.g., incidence, prevalence, outcomes analysis, survival analysis, propensity score matching) allow for patient-level analyses, while only reporting population-level data. The MetroHealth System, Cleveland OH, IRB determined research using TriNetX, in the way described here, is not Human Subject Research and therefore IRB exempt.

TriNetX is a platform that de-identifies and aggregates electronic health record (EHR) data from contributing healthcare systems, most of which are large academic medical institutions with both inpatient and outpatient facilities at multiple locations, across all 50 states in the US. TriNetX Analytics provides web-based and secure access to patient EHR data from hospitals, primary care, and specialty treatment providers, covering diverse geographic locations, age groups, racial and ethnic groups, income levels, and insurance types including various commercial insurances, governmental insurance (Medicare and Medicaid), self-pay/uninsured, worker compensation insurance, military/VA insurance among others.

Self-reported sex (female, male), race, and ethnicity data in TriNetX comes from the underlying EHR systems of the contributing healthcare systems. TriNetX maps race and ethnicity data to the following categories: (1) Race: Asian, American Indian or Alaskan Native, Black or African American, Native Hawaiian or Other, White, Unknown race; and (2) Ethnicity: Hispanic or Latino, Not Hispanic or Latino, Unknown Ethnicity.

TriNetX completes an intensive data preprocessing stage to minimize missing values. All covariates are either binary, categorical (which expands to a set of binary columns), or continuous but essentially guaranteed to exist. Age is guaranteed to exist. Missing sex values are represented using "Unknown Sex". The missing data for race and ethnicity are presented as "Unknown race" or "Unknown Ethnicity". For other variables including medical conditions, procedures, lab tests, and socio-economic determinant health, the value is either present or absent so "missing" is not pertinent.

Survival Analysis

The Kaplan-Meier analysis estimates the probability of the outcome at a respective time interval (daily time interval is used). In order to account for the patients who exited the cohort during the analysis period, and therefore should not be included in the analysis, censoring is applied.

Data Availability Statement

This study used population-level aggregate and de-identified data generated by the TriNetX Platform. Due to data privacy, patient-level data were not used and cannot be shared.

Code Availability Statement

Cohort study design and associated statistics including propensity-score matching and Kaplan-Meier survival analysis were conducted within the TriNetX Advanced Analytics Platform using built-in functions (R version 4.0.2) with significance set at p-value < 0.05 (two-sided).

© 2023 Wang L et al. *JAMA Oncol.*
