# Supplementary material for: GLP-1 Receptor Agonists and Colorectal Cancer Risk in Drug-Naive Patients With Type 2 Diabetes, With and Without Overweight/Obesity
Source: JAMA Oncol. 2023 Dec 7;10(2):256–8. doi: 10.1001/jamaoncol.2023.5573 (PMC10704339; doi:10.1001/jamaoncol.2023.5573)
Supplement: Supplement 2. — Data Sharing Statement [file jamaoncol-e235573-s002.pdf]

Data Sharing Statement

Wang. GLP-1 Receptor Agonists and Colorectal Cancer Risk in Drug-Naive Patients With Type 2 Diabetes, With and Without Overweight/Obesity. *JAMA Oncol*. Published December 07, 2023. doi:10.1001/jamaoncol.2023.5573

Data
Data available: No

Additional Information
Explanation for why data not available: This study used population-level aggregate and de-identified data generated by the TriNetX Platform. Due to data privacy, patient-level data was not used and cannot be shared.
